# Impacts of pre‐existing diabetes mellitus on colorectal cancer in a mice model

**DOI:** 10.1002/cam4.5868

**Published:** 2023-03-31

**Authors:** Yangbo Lv, Shuiquan Lin, Mingsheng Liu, Lihui Wang, Xiaoyu Wang, Li Cui, Jianguang Xu

**Affiliations:** ^1^ Department of Colorectal Surgery The Quzhou Affiliated Hospital of Wenzhou Medical University, Quzhou People's Hospital Zhejiang Quzhou China; ^2^ Shanghai Key Laboratory of Veterinary Biotechnology, School of Agriculture and Biology Shanghai Jiao Tong University Shanghai China; ^3^ Department of Gastroenterology The Quzhou Affiliated Hospital of Wenzhou Medical University, Quzhou People's Hospital Zhejiang Quzhou China

**Keywords:** colorectal cancer, diabetes mellitus, gut microbiota, T‐cell infiltration, transcriptome

## Abstract

**Background:**

Although diabetes mellitus (DM) is regarded as a risk factor of colorectal cancer (CRC), the impacts of pre‐existing DM on CRC without drug intervention remain unknown. The purpose of this study was to investigate and analyze the effects of diabetes mellitus (DM) on colorectal cancer (CRC). And, to further explore the influencing factors and the mechanisms of DM affects CRC progression.

**Methods:**

In this study, we investigated the effects of DM on CRC progression in a streptozotocin‐induced DM mice model. Furthermore, we evaluated the change of T cells levels using flow cytometry and indirect immunofluorescence. We assessed the alternation of gut microbiome and the transcriptional response using 16s rRNA sequencing and RNA‐seq.

**Results:**

Results showed that the mice survival time was significantly decreased in CRC complicated with DM group (DM‐CRC), compared with only tumor bearing mice (CRC group). Furthermore, we found that DM could affect the immune response by changing the infiltration of CD4^+^ T cells, CD8^+^ T cells and mucosal‐associated invariant T cell (MAIT) in the CRC progression. In addition, DM could induce gut microbiome dysbiosis and change the transcriptional response in CRC complicated with DM.

**Conclusion:**

For the first time, the effects of DM on CRC were systematically characterized in a mice model. Our findings highlight the effects of pre‐existing DM on CRC, and these findings should facilitate further studies in exploring and developing potentially targeted therapy for CRC in diabetic patients. Our results suggest that the effects induced by DM should be considered in the treatment for CRC complicated with DM patients.

## Graphical abstract

Graphical abstract exhibition of the effects of DM on CRC.

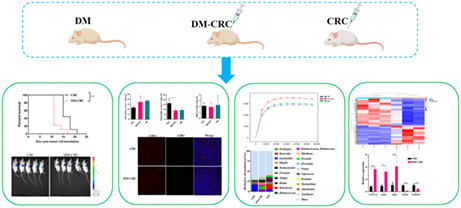

## 1 INTRODUCTION

Diabetes mellitus (DM) is a metabolic disease that has become a public health problem across the globe and is associated with substantial comorbidities and mortality.^1^ The number of DM patients is estimated to increase up to 700 million people by 2045, and the global diabetes prevalence is at a growing trend according to the International Diabetes Federation results.^2^ Cancer is widely recognized as a global common cause of mortality from noncommunicable diseases.^3^ A number of studies reported that many types of cancers complicated with DM are among the most common and complex diseases, and epidemiological data support a consistent link between DM and cancer risk, development, and mortality.^4^, ^5^

Colorectal cancer (CRC) has become the third most common cancer worldwide and is responsible for the second cause of cancer‐related death, although it is a preventable disease.^6^ Studies prove that the pre‐existing DM is considered to be an independent risk factor that promotes CRC carcinogenesis and an exacerbation factor.^7^ Previous study found a modest association between DM and CRC was only present among men, but not women.^8^ CRC patients with DM may have a worse outcome than the non‐diabetic counterparts,^9^ and people a diabetes diagnosis before the age of 50 have nearly a twofold increased risk for developing CRC.^10^ Studies found that insulin treatment and some other glucose‐lowering treatments themselves are also related with cancer risk and mortality, including CRC.^11^, ^12^ The majority of researches related to the association between pre‐existing DM and cancer are retrospective studies, with only a few cohort studies.^1^, ^13^ It is clear that there are many complex factors related with DM and cancer development, it is speculated that shared risk factors present at the time of DM emergence contribute the onset of the oncogenic milieu.

Although DM is considered as a risk factor of cancer including CRC, the impacts of pre‐existing DM on CRC without drug intervention remain unclear. Thus, in the present study, we evaluated systematically the effects of preexisting DM on CRC progression using a streptozotocin‐induced DM mice model. We further evaluated the alternation of gut microbiota, T‐cell response, and the changes in transcript levels from tumor tissues in CRC complicated with DM and without DM. Our results help address the challenge of developing CRC complicated with DM treatment plan.

## 2 MATERIALS AND METHODS

### 2.1 Cell culture and antibodies

The mouse CRC cell lines CT26 were obtained from the American Type Culture Collection (ATCC), and cultured in RPMI 1640 (Gibco) with 10% FBS (Australia origin) and 1% penicillin/streptomycin (Gibco) in a humidified atmosphere with 5% CO_2_ at 37 °C. The antibodies for FCM analysis including anti‐CD4, anti‐CD8, anti‐CD3, anti‐TCR beta, and MR1 were bought from Becton Dickinson (BD). The antibodies (anti‐CD4, anti‐CD8, anti‐rabbit IgG, anti‐mouse IgG) for indirect immunofluorescence (IF) analysis were bought from Thermo Fisher Scientific (Waltham).

### 2.2 Mice model of CRC complicated with DM

Female BALB/c mice (4–6 weeks old) were bought from SPF Biotechnology Co., LTD (Beijing). Mice received an intraperitoneal injection of streptozotocin or citrate buffer (Sigma‐Aldrich) with 130 mg/kg body using a 29‐G needles.^14^ The STZ solution should be dissolved in sodium citrate buffer (pH 4.5), and prepared fresh immediately before injection and injected within 5 min of being dissolved.^15^ The blood glucose level was monitored using a blood glucose meter, and it was regarded as a valid DM mice model when the glucose concentration reached 12 mmol/L.^16^ The CT26 cell suspensions were prepared, and each mouse was subcutaneously injected with 1 × 10^6^ CT26 cells or phosphate‐buffered saline (PBS) buffer.

### 2.3 Tumor measurement

The tumor size was tested using a digital caliper, and tumor volume is calculated as (Length × width^2^)/2.^17^ For ethical reasons, mice were killed when the tumor volume reached 2000 mm^3^. Imaging of CT26‐bearing mouse was detected using the IVIS Spectrum imaging system (IVIS Spectrum).

### 2.4 Flow cytometric analysis

The levels of CD4^+^ cells, CD8^+^ T cells, and mucosal‐associated invariant T cell (MAIT) cells were detected by flow cytometry (FCM). The samples were prepared as previously reported.^18^ Briefly, the mice were killed, and single‐cell suspensions from the blood and tumor tissues of different groups were prepared, following three washes with PBS, incubated for 30 min in the dark at 4°C with their respective primary antibodies, and then washed three times with staining buffer. The isotype‐matched mAbs was used for control staining. Finally, the cells were resuspended in 200 μL staining buffer for FCM analysis (Beckman CytoFLEX).

### 2.5 Indirect immunofluorescence

The tumor tissue samples collected from different groups were fixed. IF was used to detect T‐cell infiltration in the tumor microenvironment. Both primary and secondary antibodies associated with T‐cell detection were bought from Thermo Fisher Scientific. The 4′, 6‐diamidino‐2‐phenylindole (DAPI) was bought from (Beyotime Biotechnology). Finally, the slides were washed with PBST and imaged under a fluorescence microscope.

### 2.6 Samples preparation and DNA extraction

The fecal samples from the DM, DM‐CRC, and CRC groups were collected, and DNA were extracted using a Microbiome DNA Purification Kit (Thermo Fisher Scientific) according to the manual protocols. A NanoDrop ND‐1000 spectrophotometer (Thermo Fisher Scientific) was used to test the concentration of DNA. The DNA was stored at −80°C until further experiments.

### 2.7 16S rRNA sequencing

The 16S rRNA gene was sequenced using the Illumina MiSeq platform (Illumina). 16S rRNA gene (hypervariable V3–V4 region) were amplified with the primer pair F (ACTCCTACGGGAGGCAGCA) and R (GGACTACHVGGGTWTCTAAT). The raw data were processed as previously reported.^19^ The metagenomeSeq was analyzed in R using metagenomeSeq package. Venn diagram was created according to the genescloud platform (https://www.genescloud.cn).

### 2.8 RNA‐seq library preparation and sequencing

Total RNA from tumor tissues in different groups was extracted and purified RNA was used for cDNA library construction according to the Collibri Stranded RNA Library Prep Kit manufacturer's instructions (Thermo Fisher Scientific). RNA‐seq used the next‐generation sequencing to analyze gene expression. The data were processed and analyzed using HISAT2 software, DESeq package, topGO, and clusterProfiler.

### 2.9 RNA extraction and quantitative real‐time PCR experiments

Total RNA was extracted from tissues, and cDNA was synthesized using PrimeScript™ II 1st Strand cDNA Synthesis Kit (Takara). The quantitative real‐time PCR (qPCR) was carried out using TB Green® Fast qPCR Mix (Takara) for 30 s at 95°C, followed by 40 cycles at 95°C for 5 s, 60°C for 10 s. The data were analyzed using the 2^−ΔΔCt^ method. All of the qRT‐PCR amplifications were performed in triplicate and were repeated three times. The primers used are listed in Table S1.

### 2.10 Statistical analysis

Student's *t*‐test was used to compare tumor burden. The log‐rank (Mantel‐Cox) test was used to analyze survival difference. GraphPad Prism 8 (GraphPad Software) was used to create chart and common data analysis. A value of *p* < 0.05 was considered significant.

## 3 RESULTS

### 3.1 DM promotes CRC progression in vivo

We evaluated the effect of DM in CRC progression using a streptozotocin‐induced DM mice model.

**FIGURE 1:**
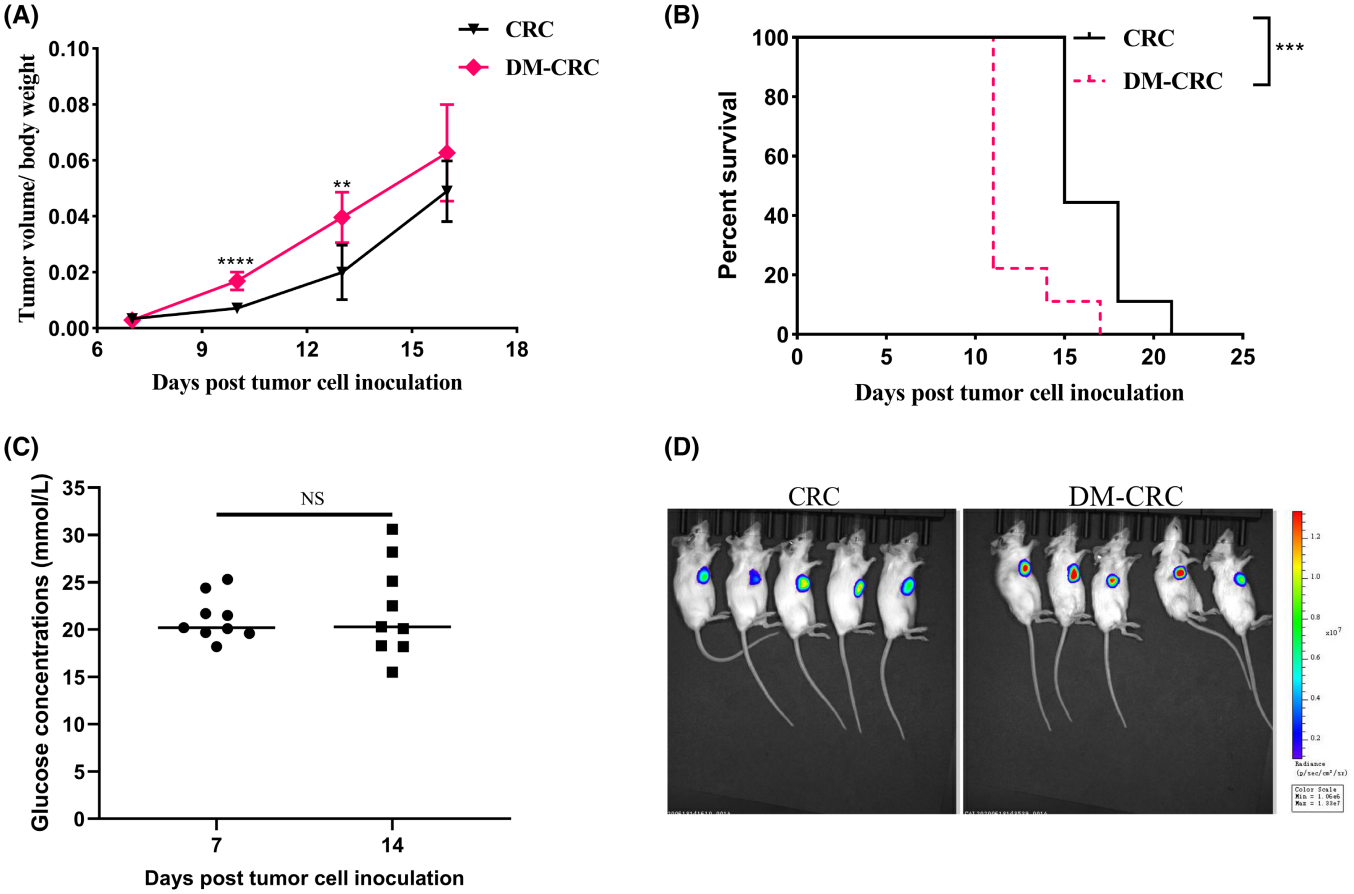
The pre‐existing DM promotes the CRC progression. (A) The calculation of ratio of tumor volume and body weight. (B) The survival curve of the CRC and DM‐CRC groups. (C) The assessment of glucose concentrations of streptozotocin‐induced DM mice. (D) Fluorescence intensity in streptozotocin‐induced mice after tumor cell inoculation detected using an IVIS imaging system. NS: no significant difference, **p < 0.01, ***p < 0.001, ****p < 0.0001. (CRC, *n* = 9; DM‐CRC, *n* = 9). These experiments were independently repeated three times with similar results. Error bars stand for the mean ± SD.

The results indicated that there was a higher ratio of mouse tumor volume/body weight in the DM‐CRC group compared to the CRC group (Figure 1A). Furthermore, we found that the survival in the DM‐CRC group significantly decreased compared to the CRC group (Figure 1B). The high‐glucose level remained unchanged over time (Figure 1C). According to in vivo imaging data, no metastasis was found, but the signal intensity was significantly increased in the DM‐CRC group compared to the CRC group (Figure 1D). These results showed that DM promoted CRC progression and might be a key risk factor for CRC.

### 3.2 DM mediates specific immune response in CRC complicated DM mice model

**FIGURE 2:**
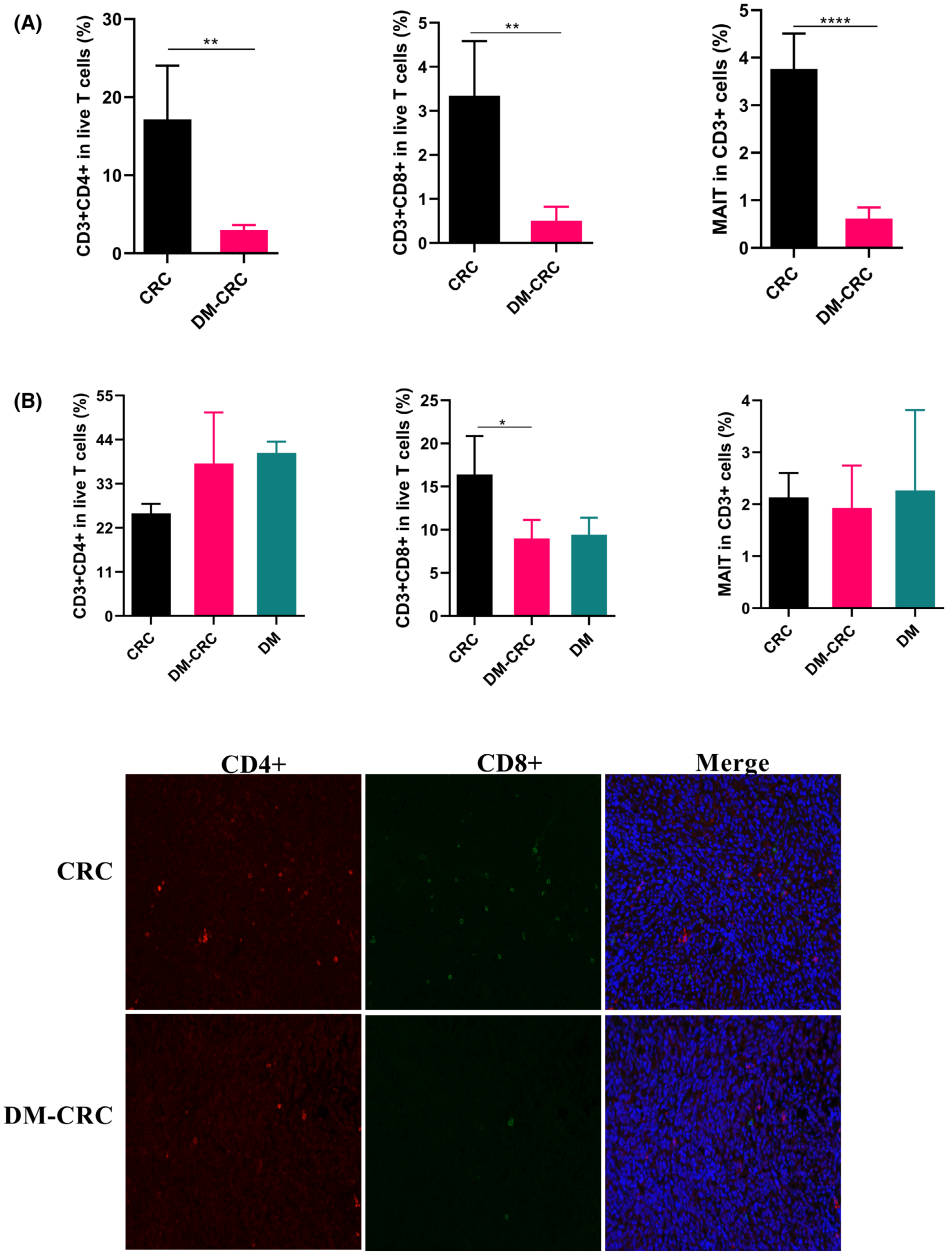
The pre‐existing DM changes the levels of CD4^+^ T, CD8^+^ T, and MAIT cells in tumor. (A,B) The levels of CD4^+^ T, CD8^+^ T, and MAIT cells in tumor (A) and in PBMC (B) were analyzed using FCM. (C) The infiltration of CD4^+^ T cells (red) and CD8^+^ T cells (green) in tumor tissue were tested using IF. *p < 0.05, **p < 0.01, ****p < 0.0001 (CRC, *n* = 5; DM‐CRC, *n* = 4, DM, *n* = 5). These experiments were independently repeated three times with similar results. Error bars stand for the mean ± SD.

It is well known that the infiltrations of T cells in tumor environment have key roles cancer immunotherapy,^20^ and MAIT cells promote tumor initiation, progression, and metastasis.^21^ In this study, we investigated whether CD3^+^CD4^+^ T cells, CD3^+^CD8^+^ T cells, and MAIT cells are related to CRC complicated with DM. Here, we analyzed the proportions of CD3^+^CD4^+^ T cells, CD3^+^CD8^+^ T cells, and MAIT cells from different groups in the tumor tissues and in the peripheral blood mononuclear cell (PBMC) using FCM. Our result showed that the proportions of CD3^+^CD4^+^, CD3^+^CD8^+^, and MAIT cells in the DM‐CRC group in tumor significantly decreased compared with that in the CRC group (Figure 2A). The levels of T cells in PBMC were also analyzed. The proportion of CD3^+^CD8^+^ T cells in the PBMC in the DM‐CRC group significantly decreased compared with the proportion of CD3^+^CD8^+^ T cells in the CRC group (Figures 2B), while no obvious difference in the levels of CD3^+^CD4^+^ T cells and MAIT cells in the PBMC was found among different groups (Figure 2B). Besides, we analyzed the levels and distributions of CD3^+^CD4^+^ T and CD3^+^CD8^+^ T cells in the tumor microenvironment using IF. Results showed that the number of CD3^+^CD4^+^ T and CD3^+^CD8 ^+^ T cells in tumor from the DM‐CRC group was less than that in the CRC group (Figure 2C). These results suggested that the DM‐promoted CRC progression might be caused in part by influencing T‐cell levels and infiltrations.

### 3.3 DM alters the gut microbiota in CRC complicated DM mice model

**FIGURE 3:**
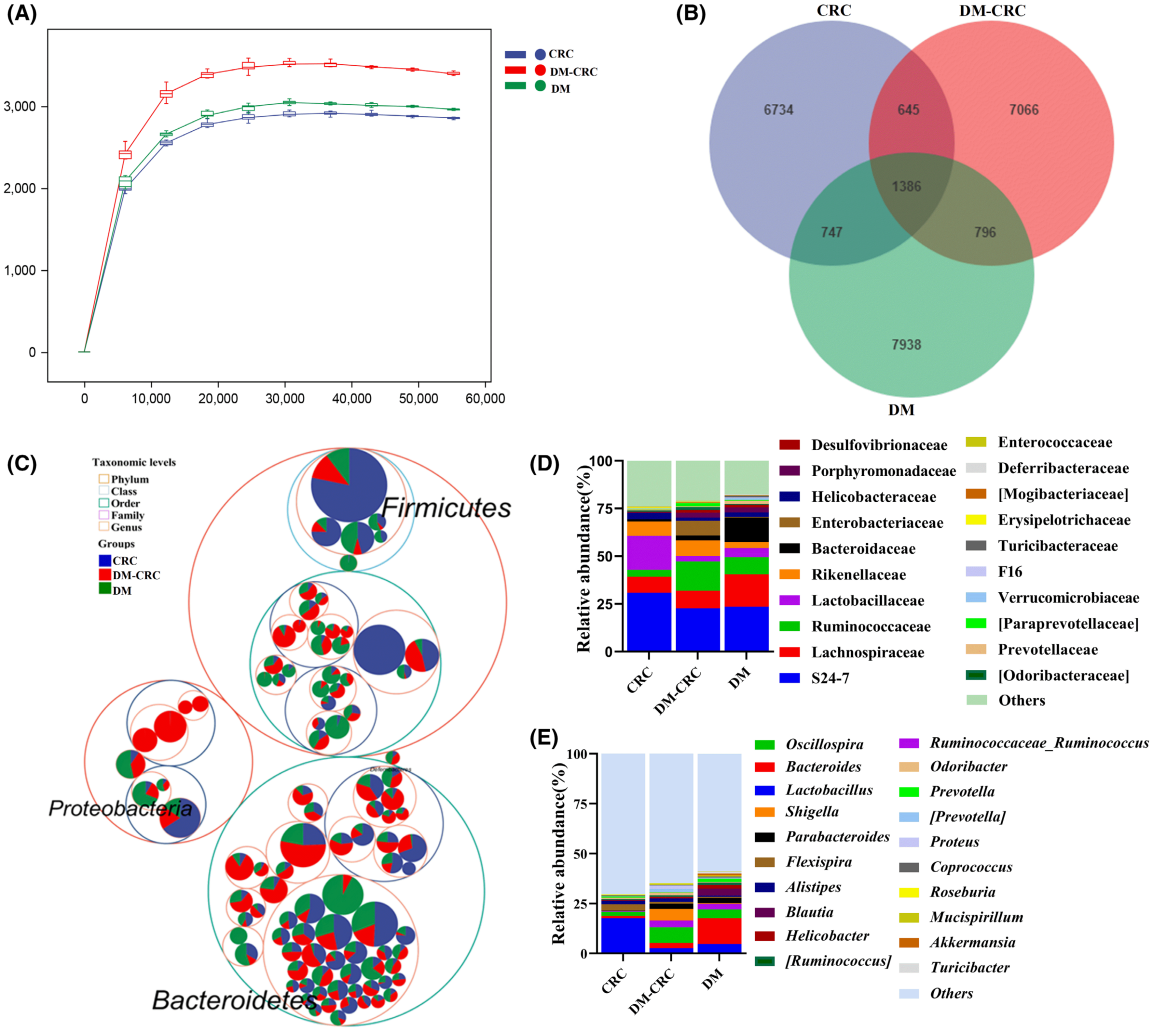
DM alters the gut community in CRC complicated DM mice model. (A) Rarefaction curve of the estimated number of genera using the Chao1 method. The abscissa represents reads number and the ordinate represents Chao1. (B) A Venn diagram displaying the overlaps among different groups. (C) Taxonomic differences are based on 16S rRNA seq. Taxonomic composition visualized by circular packing. The largest circles represent phylum level, and the inner circles represent class, family, and genus. (D,E) Total gut bacterial relative abundance at the taxonomic rank of family (D) and genus (E) of the CRC, DM‐CRC, and DM groups (CRC, *n* = 5; DM‐CRC, *n* = 4, DM, *n* = 5).

Dysbiosis of the gut microbiota has been involved in the development of CRC, and also linked to the development of DM. Our results showed that DM promoted CRC progression (Figure 1). But the role of gut microbiota in CRC complicated with DM without any drug intervention remains to be fully investigated. Here, we analyzed the gut microbiota difference among DM, CRC, and DM‐CRC using 16s rRNA seq. Rarefaction analysis showed that richness in each group approached saturation (Figures 3A and S1A,B). The analysis of alpha‐diversity of the gut microbiome at the baseline did not differ significantly among different groups (Figure S1C and Table S2). The Venn diagram showed that 1386 ASVs were common to all the three groups, while 6734 ASVs and 7066 ASVs were unique to the CRC and DM‐CRC groups, respectively (Figure 3B).

We further identified the bacterial composition and alterations of the bacterial microbiome in the CRC, DM‐CRC, and DM groups. Proteobacteria, Bacteroidetes, and Firmicutes are the main bacterial community in all the groups (Figure 3C). The composition of different groups at the phylum, family, and genus levels were analyzed. The bacteria composition in the DM‐CRC group was different from the DM and CRC groups (Figure 3D,E and S1D). At the genus level, *Lactobacillus* in the DM‐CRC group decreased compared with the DM and CRC groups, while *Oscillospira* increased in the DM‐CRC group compared with the DM and CRC groups (Figure 3E).

**FIGURE 4:**
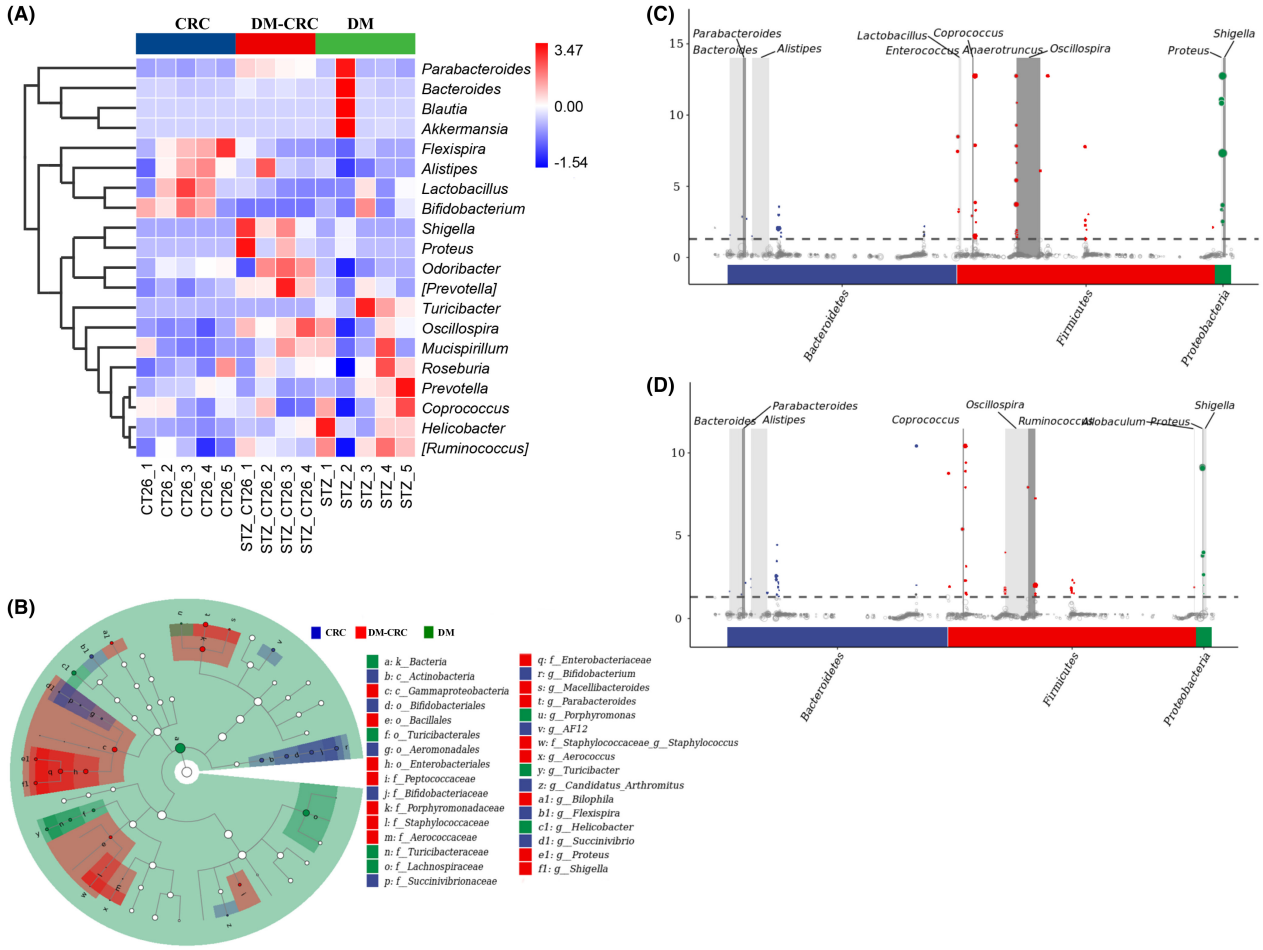
DM results in gut microbiota dysbiosis in a CRC complicated DM mice model. (A) Heatmap of the relative genus abundances of differential bacteria for each sample in three different groups. (B) The LEfSe cladogram, representing gut microbial structure and their predominant bacteria, revealed the greatest differences in taxa among different groups. (C,D) Manhattan plots showing the abundance enrichment in the DM‐CRC group compared with the CRC group (C) and DM group (D), respectively.

The top 20 genera of each group are displayed in Figure 4A. Furthermore, we performed linear discriminant analysis (LDA) effect size (LEfSe) and metagenomeSeq analysis to show significantly different bacteria among different groups. In the present study, an LDA score of >2 was used as the cutoff value, and the cladogram representing gut microbial structure and their respectively predominant bacteria showed the most differences in taxa among different groups (Figure 4B). Fifty‐eight different ASVs were found to be enriched in the DM‐CRC group compared to the CRC group. These ASVs mainly distributed in *Parabacteroides*, *Bacteroides*, *Alistipes*, *Lactobacillus*, *Enterococcus*, *Coprococcus*, *Anaerotruncus*, *Oscillospira*, *Proteus*, and *Shigella* at the genus level (Figure 4C). When compared with the DM group, there are about 57 ASVs that are mainly enriched in the DM‐CRC group, and these ASVs mainly belonged to *Bacteroides*, *Parabacteroides*, *Alistipes*, *Coprococcus*, *Oscillospira*, *Ruminococcus*, *Allobaculum*, *Proteus*, and *Shigella* (Figure 4D). These results suggested that streptozotocin‐induced DM could change the composition and abundance of gut bacteria.

### 3.4 DM changes the tumor transcriptional response

**FIGURE 5:**
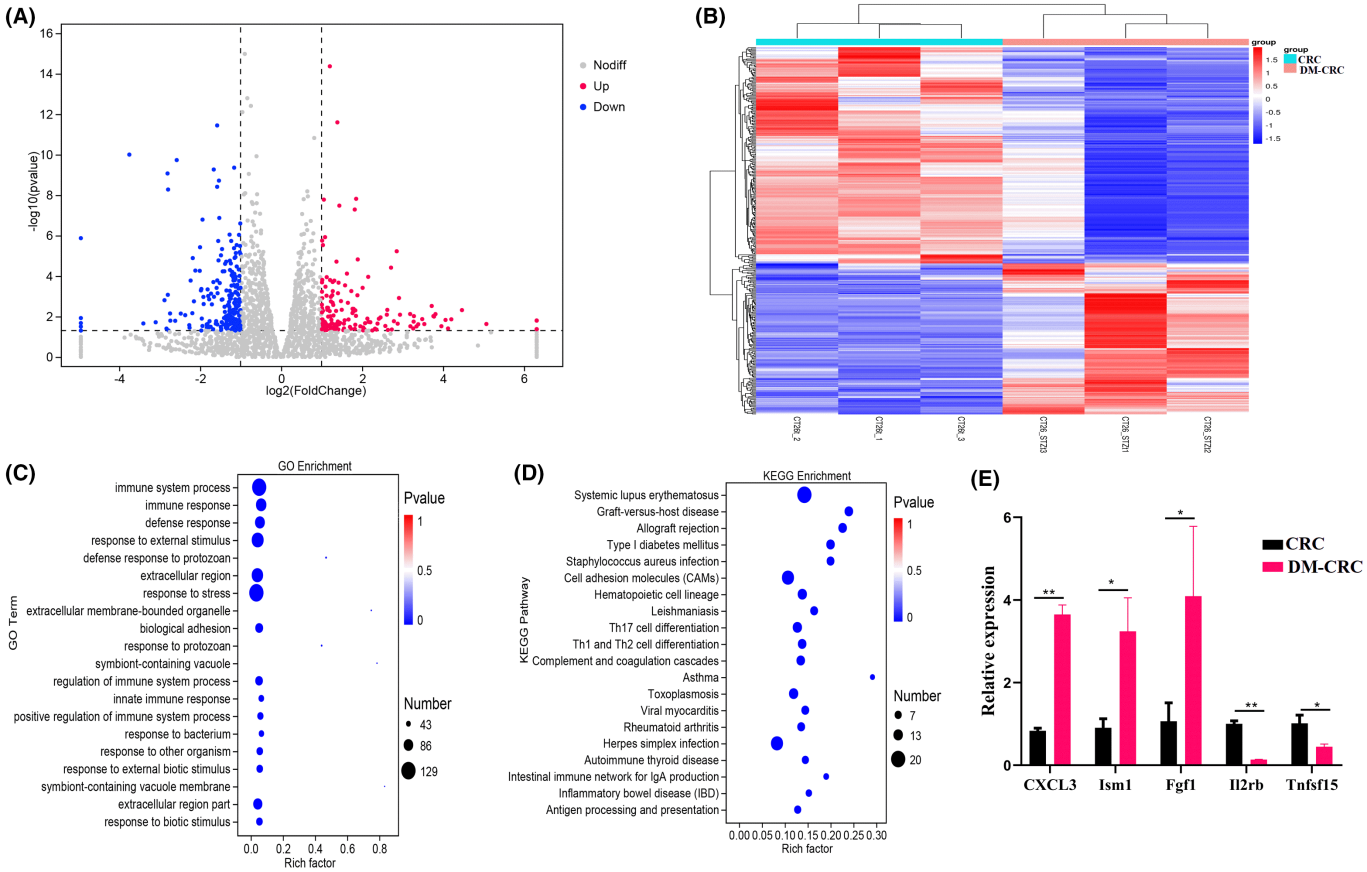
Streptozotocin‐induced DM alters the transcriptional response in tumor site. (A) Volcano plots of DEGs between the DM‐CRC and CRC groups. Red dots indicate upregulation. Blue dots indicate downregulation. Gray dots indicate no significant difference. (B) Heatmap of differential expression genes in the DM‐CRC and CRC groups. (C) Top 20 GO term assignments for DEGs between the DM‐CRC and CRC groups. (D) Top 20 KEGG pathways of DEGs. The color of nodes indicated *p* value and the size of nodes showed the count of DEGs enriched. (E) Further validation of the RNA seq using qRT‐PCR. * *p* < 0.05, ** *p* < 0.01 (CRC, *n* = 3; DM‐CRC, *n* = 3). Error bars stand for the mean ± SD.

Although DM affects the CRC progression and is regarded as a risk factor in CRC, the molecular mechanisms that CRC complicated with DM remains unclear. Furthermore, we analyzed the differential gene expression profiles using RNA‐seq. In total, 360 genes were found to be expressed differently in the DM‐CRC group compared with the CRC group (Figure 5A). Heat map showed a significant difference in the transcripts between the DM‐CRC and CRC groups (Figure 5B). Furthermore, we found that DEGs between the DM‐CRC and CRC groups were mainly involved in immune system process, response to external stimulus, response to stress, systemic lupus erythematosus, cell adhesion molecules, Th1, Th2, and Th17 cells differentiation (Figure 5C,D). DEGs were randomly selected for further validation by qRT‐PCR, and the results were consistent with the RNA‐seq data (Figure 5E).

## 4 DISCUSSION

Cancer and DM are leading causes of mortality and morbidity in the general population globally.^22^ Numerous epidemiological studies have showed that there is a relationship between DM and CRC,^23^, ^24^ but only a few researches have attempted to systemically elucidate the effects of DM on CRC. In this study, we found DM can decrease the survival time of CRC (Figure 1), which is consistent with most epidemiological findings. Our results suggested DM may play important roles in CRC development and progression.

MAIT cells are innate‐like T lymphocytes which can bridge the innate and adaptive immune systems, and MAIT cells have lymphokine‐activated killer activity. MAIT cell deficiency is related to the degree of CRC progression.^25^ According to our data, DM reduce the level of MAIT in tumor. The changed distribution of MAIT may be associated with the gut dysbiosis. The diversity of MAIT may play different roles in cancer progression. More studies should focus how DM changed the level of MAIT. WDM decreased the level of CD4^+^ and CD8^+^ T cells in tumor (Figure 2A). T‐cell‐mediated immune response, particularly T‐cell infiltration in tumor tissues, play important roles in CRC prognosis.^26^ Our results suggested that the decrease in T‐cell infiltration may contribute to the DM‐promoting cancer progression, and T‐cell levels of tumor microenvironment should be carefully evaluated in clinical therapy.

It is well known that the gut bacteria play important roles both in DM and CRC progression. Increasing studies indicated that dysbiosis of the bacterial microbiota is closely related to CRC^27^ and associated with DM.^28^ As previously reported, some bacteria species are related to CRC initiation, progression and metastasis, including *Fusobacterium nucleatum*, *Escherichia coli*, *Peptostreptococcus anaerobius*.^29^ The bacterium *F. nucleatum* may promote CRC development by regulating T‐cell‐mediated adaptive immunity.^30^ In our study, we found that both streptozotocin‐induced DM and tumor altered the gut bacterial composition and abundance (Figures 3 and 4). Some specific probiotics, such as *Lactobacillus* and their metabolites are involved in the suppressing the growth of CRC cells.^31^ The abundance of *Lactobacillus* was significantly reduced in CRC complicated with DM compared with only the CT26 tumor‐bearing mouse group (Figure 3E), which suggested that *Lactobacillus* may play important roles in the progression of CRC complicated DM. Prebiotic combined therapy showed improvements in fasting blood glucose, glucose tolerance, and insulin resistance by modulating gut microbiota in a streptozotocin‐induced diabetic mice model.^32^ *Bacteroides* plays a beneficial role in glucose metabolism in humans and experimental animals, and *Bacteroides* was significantly decreased in the DM‐CRC group compared to that in the DM group, which suggested that *Bacteroides* may be involved in CRC‐complicated DM. To our knowledge, this is the first time to characterize the specific bacteria profile, which contribute to the association of CRC complicated with DM. We are also currently studying the causal relationships between gut microbiota dysbiosis and disease progression in mice model and clinical cohorts.

Numerous of epidemiological studies have indicated that DM is associated with the increased risk for CRC, but the mechanism remains unclear. In this study, we investigated the underlying molecular mechanism of the link between CRC and DM using RNA‐seq. Our results showed that a total of 360 DEGs, including 148 upregulated and 212 downregulated DEGs were detected between the DM‐CRC and CRC groups. Among these DEGs, Fgf1 belonged to fibroblast growth factors (Fgfs) family, which is heparin‐binding growth factors, it has been confirmed to be involved in the invasion and metastasis of tumor and also has a great potential for the treatment of DM.^33^, ^34^ Although it is well known that Fgfs family is associated with both CRC and DM, but the role of Fgfs in CRC complicated with DM remains unclear. Our mice model data showed that Fgf1 was significantly increased in the CRC complicated with DM group compared with the only CRC tumor group (Figure 5E). These findings suggest Fgr1 is a potential target for the treatment of CRC complicated with DM. Chemokines were associated with the recruitment of T cells to the tumor site,^35^ and both the abnormal expression of CXCL3 and the increase in T‐cell infiltration were found in CRC complicated with DM in this study (Figure 5E). The correlations between the levels of CXCL3 and T‐cell infiltration need further validation. In our future studies, we will also focus on the association and link between these DEGs and gut microbiota and also T‐cell response.

## 5 CONCLUSION

In conclusion, we found the pre‐existing DM promotes CRC progression in a mice model. Furthermore, we found that DM changed tumor microenvironment and the gut microbiome. Our findings highlight the effects of pre‐existing DM on CRC, and these findings should facilitate further studies in exploring and developing potentially targeted therapy for CRC in diabetic patients

## AUTHOR CONTRIBUTIONS

**Yangbo Lv:** Conceptualization (lead); data curation (lead); formal analysis (equal); writing – original draft (lead); writing – review and editing (equal). **Shuiquan Lin:** Data curation (equal); formal analysis (equal); methodology (equal). **Mingsheng Liu:** Data curation (equal); formal analysis (equal); investigation (equal); methodology (equal). **Lihui Wang:** Data curation (equal); formal analysis (equal); investigation (equal); methodology (equal). **Xiaoyu Wang:** Data curation (equal); formal analysis (equal); methodology (equal). **Li Cui:** Conceptualization (lead); funding acquisition (lead); supervision (equal). **Jianguang Xu:** Conceptualization (lead); investigation (equal); supervision (lead).

## FUNDING INFORMATION

This work was sponsored by grants from the Shanghai Science and Technology Innovation Action Plan (21140901400).

## CONFLICT OF INTEREST STATEMENT

The authors declare that there is no conflict of interest that could be perceived as prejudicing the impartiality of the research reported.

## ETHICS STATEMENT

The animal experiments in our study were approved by the local Animal Ethical Committee (SV20210316‐01). After approval, the experiments were performed according to the Guidelines on the Humane Treatment of Laboratory Animals. The study did not involve human trials, so no informed consent was required for the study.

## Supporting information


**Data S1.** Supporting Information.

## Data Availability

All the sequencing data have been deposited in the Sequence Read Archive (SRA), under the accession number SRP351186 and SRP351836.
