# Correction to “Impacts of pre‐existing diabetes mellitus on colorectal cancer in a mice model”

**DOI:** 10.1002/cam4.6872

**Published:** 2024-01-18

Lv Y, Lin S, Liu M, et al. Impacts of pre‐existing diabetes mellitus on colorectal cancer in a mice model. *Cancer Med*. 2023;12:11641–11650. doi:10.1002/cam4.5868

In the correspondence section, the affiliations of Jianguang Xu and Li Cui were misplaced. The corrected affiliations appear below.

**Correspondence**

Jianguang Xu, Department of Gastroenterology, The Quzhou Affiliated Hospital of Wenzhou Medical University, Quzhou People's Hospital, Quzhou, Zhejiang, China. Email: 12689513@qq.com

Li Cui, Shanghai Key Laboratory of Veterinary Biotechnology, School of Agriculture and Biology, Shanghai Jiao Tong University, Shanghai, China. Email: lcui@sjtu.edu.cn

We apologize for this error.

