# Supplementary material for: Impacts of pre‐existing diabetes mellitus on colorectal cancer in a mice model
Source: Cancer Med. 2023 Mar 31;12(10):11641–50. doi: 10.1002/cam4.5868 (PMC10242856; doi:10.1002/cam4.5868)
Supplement: Supplementary file 1 — Data S1. Supporting Information. [file CAM4-12-11641-s001.docx]

**Supplement Figure and legends**

**
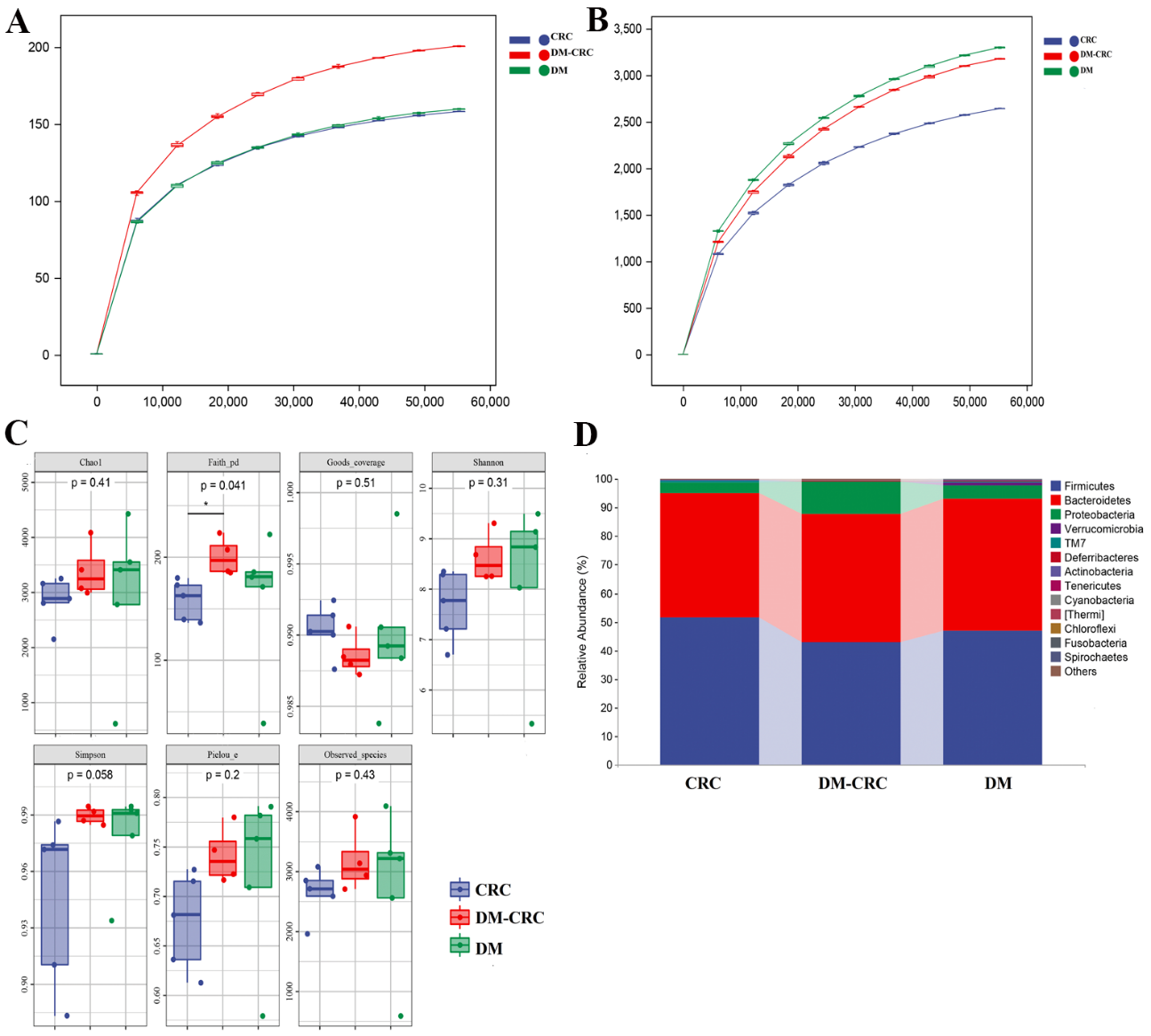
**

**Figure S1.** **(A)** Rarefaction curve of the estimated number of genera using the Faith_pd method. The abscissa represents reads number and the ordinate represents Faith_pd. **(B)** Rarefaction curve of the estimated number of genera using the Observed_species method. The abscissa represents reads number and the ordinate represents Observed_species. **(C)** Alpha diversity boxplot in the gut microbial community based on the Chao1, Faith_pd, Goods_coverage, Observed_species, Pielou_e, Shannon and Simpson. **(D)** Average of compositions and relative abundance of bacterial at the taxonomic rank of phylum in different groups. *P* values was determined by Kruskal-Wallis test.

**Supplement table**

**Table S1** Primer sequences used in qRT-PCR analysis. Note: F indicates forward primer, R means reverse primer

| Gene name | Primer sequence (5’→3’) | Amplication length |
| --- | --- | --- |
| *CXCL3*-F | CAGCCACACTCCAGCCTA | 113 bp |
| *CXCL3*-R | CACAACAGCCCCTGTAGC |  |
| *Ism1*-F | AGAGCAGCCAGAGTATGATTCC | 189 bp |
| *Ism1*-R | GCCGCTGTCCTGAAAGTATCT |  |
| *Fgf1*-F | ACCAAACTATACAGCCGACAAG | 108 bp |
| *Fgf1*-R | AGGGATGAGGTTAAACAGAGTGT |  |
| *Il2rb*-F | CCAATGTCTCTTGCATGTGGA | 149 bp |
| *Il2rb*-R | CCCGAGGATCAGGTTGCAG |  |
| *Tnfsf15*-F | AGTCCCAGTGGAAGTGCTG | 135 bp |
| *Tnfsf15*-R | GTGCTAAGTCCTGCGAGGAT |  |
| *Actin*-F | AGAGGGAAATCGTGCGTGAC | 195 bp |
| *Actin*-R | CCATACCCAAGAAGGAAGGCT |  |

**Table S2** **Alpha-diversity indexes in the gut of adults from different groups**

| Sample | Chao1 | Faith_pd | Goods_coverage | Observed_species | Pielou_e | Shannon | Simpson | Group |
| --- | --- | --- | --- | --- | --- | --- | --- | --- |
| CT26_1 | 2814.29 | 139.359 | 0.991399 | 2592.1 | 0.636174 | 7.21415 | 0.883281 | CRC |
| CT26_2 | 3254.19 | 179.712 | 0.99026 | 3086.4 | 0.715333 | 8.29193 | 0.971719 |  |
| CT26_3 | 2150.68 | 136.341 | 0.992441 | 1961.8 | 0.612895 | 6.70381 | 0.910458 |  |
| CT26_4 | 2891.77 | 162.642 | 0.990021 | 2713.6 | 0.681694 | 7.77539 | 0.974154 |  |
| CT26_5 | 3163.3 | 172.792 | 0.987595 | 2852.9 | 0.727577 | 8.35128 | 0.986594 |  |
| STZ_CT26_1 | 3080.72 | 186.709 | 0.990598 | 2939.2 | 0.716833 | 8.25878 | 0.987225 | DM-CRC |
| STZ_CT26_2 | 2997.98 | 185.063 | 0.988481 | 2710.4 | 0.723155 | 8.24706 | 0.984728 |  |
| STZ_CT26_3 | 3417.05 | 207.109 | 0.987233 | 3140.7 | 0.747686 | 8.68576 | 0.991923 |  |
| STZ_CT26_4 | 4091.28 | 223.75 | 0.987982 | 3918.3 | 0.779833 | 9.30809 | 0.994722 |  |
| STZ_1 | 3553.67 | 181.011 | 0.98839 | 3316.4 | 0.782065 | 9.14656 | 0.992823 | DM |
| STZ_2 | 621.184 | 38.5804 | 0.998512 | 593.8 | 0.579526 | 5.33964 | 0.933887 |  |
| STZ_3 | 2782.14 | 171.378 | 0.990551 | 2563.7 | 0.709269 | 8.03176 | 0.979144 |  |
| STZ_4 | 4432.52 | 222.229 | 0.983796 | 4094 | 0.791372 | 9.4959 | 0.994467 |  |
